# Beyond the Flames: Public Health Management and Policy Implications From the Wang Fuk Court Fire Disaster in Hong Kong

**DOI:** 10.34172/ijhpm.9671

**Published:** 2026-05-24

**Authors:** Eliza Lai-Yi Wong, Eng-Kiong Yeoh, Samuel Yeung-Shan Wong

**Affiliations:** The Jockey Club School of Public Health and Primary Care, Faculty of Medicine, The Chinese University of Hong Kong, Hong Kong SAR, China.

## Introduction

 On November 26, 2025, a catastrophic fire occurred during large-scale building maintenance at Wang Fuk Court, a government-subsidised residential estate in Tai Po, Hong Kong (HK). The blaze rapidly spread via renovation scaffolding and engulfed seven of the eight high-rise residential towers. The disaster affected around 2000 residents and resulted in 168 fatalities, including the death of one firefighter, and 79 non-fatal injuries as of January 15, 2026, making it one of the city’s deadliest urban fires in recent decades and a stark reminder of the vulnerabilities of high-density living.^1-3^ Beyond its immediate mortality and injury burden, the incident illustrates how housing safety and building regulation function as upstream health policy mechanisms, shaping population exposure to preventable risks. In HK, the Mandatory Building Inspection Scheme is intended as a preventive regulatory instrument to mitigate hazards in ageing buildings; however, its effectiveness has direct implications for injury prevention, emergency healthcare demand, and health equity. Buildings aged 30 years or older (excluding domestic buildings of three storeys or fewer) that receive statutory notices must undergo inspection and repairs.^4^ Additionally, buildings typically reach their design working life at around 50 years, at which point further structural and safety evaluations become increasingly critical.^4^ This tragedy reflects systemic challenges in the urban environment: high-density housing, ageing buildings, social inequities and gaps in regulatory enforcement. It underscores the need for housing and safety regulators, health authorities, and policy-makers to adopt integrated governance approaches that treat building safety as a determinant of health and to align regulatory oversight with health system preparedness and post-disaster recovery strategies that address both physical and psychosocial inequities.

## Understanding Urban Fire Safety in Building Maintenance Through the 4M1E Lens

 This tragedy illustrates risks that are not confined to a single city, but may arise in urbanised regions wherever high-density environments intersect with systemic vulnerabilities. As the incident remains under investigation, the 4M1E framework is applied here to illustrate common risk factors associated with fire incidents in high-density housing, rather than to determine incident-specific causation.^5,6^ *Man-related* factors, including unsafe work practices and delayed evacuation, point to the importance of contractor training, site supervision, and emergency preparedness for both workers and residents. *Machine-related* issues, such as obstructed escape routes and compromised access points, highlight gaps in the enforcement of fire-safety design standards and routine inspection regimes. *Material-related *risks, including the use of combustible construction materials and inadequate fire-resistant barriers during renovation works, underscore the need for tighter regulatory controls on approved materials and compliance monitoring. *Method-related* factors, such as insufficient maintenance practices and ineffective operation of fire protection systems, suggest weaknesses in inspection protocols, accountability mechanisms, and coordination during building maintenance. Finally, *Environmental *conditions, including dry weather and wind, further amplify fire risk in dense urban settings and point to the value of adaptive risk management during high-risk periods. Taking together, these interacting risk factors illustrate how systemic vulnerabilities in building maintenance and regulation can combine to produce severe health consequences. Reinforcing building safety as an upstream determinant of population health is therefore important.

## Mental Health, Vulnerability, and Responders

 Fire disasters cause lasting physical, mental, and social harm. Survivors face stressors such as injury, bereavement, displacement, routine disruption, and financial strain, with risk amplified for vulnerable groups.^7,8^ Residents in subsidized housing – older adults, persons with chronic illness, low-income households, foreign workers, and children – are especially at risk. Smoke exposure, medication interruption, sleep disruption, and sudden relocation can lead to rapid decline, while children face long-term emotional and behavioural effects.^9,10^ Early identification and timely care are critical. Firefighters and other respondents also face hazards, including respiratory and cardiovascular complications, musculoskeletal injury, and post-traumatic stress disorder.^11^ Post-disaster health programmes should provide ongoing monitoring, mental health assessment, and access to evidence-based care.

## Early Response and Health System Integration

 The HK Government responded swiftly to the Wang Fuk Court fire with several emergency and recovery measures. These included emergency financial assistance of HK$100 000 as a living allowance to each affected household, the assignment of designated social workers to provide follow-up support, and the allocation of 500 units for transitional housing and over 2000 units as longer-term accommodations.^12^ Medical and psychosocial support were provided through the mobilisation of a multidisciplinary team coordinated by the relevant government units.^13,14^ Public donation campaigns, along with volunteers and care teams’ mobilisation, enabled the timely distribution of essential resources to affected residents.^15^ To address systemic accountability, the government announced an independent committee of inquiry to investigate the causes and rapid spread of fires and formulate future preventive measures.^16^ These efforts provide a critical foundation for building back better – strengthening resilience, improving safety standards and ensuring that lessons learned translate into long-term systemic reforms.

## Build Back Better: Equity, Governance and Community Resilience

 Recovery extends beyond physical reconstruction and offers an opportunity to address inequities and strengthen system resilience. In line with World Health Organization (WHO) guidance, post-fire recovery should prioritise equity, psychosocial support, integrated health services, and strong governance, with particular attention to vulnerable populations.^17^ To enhance policy relevance, recovery actions can be considered across short, medium, and long-term.

 In the short term, recovery policies must prioritise vulnerable groups through timely access to temporary housing, financial assistance, and psychosocial support. However, relocation outside their familiar neighbourhoods may disrupt social networks and access to essential services. *Assigning a dedicated social worker or public health practitioner as a resource navigator* can help coordinate housing, financial, health and community services, with relatively modest staffing and administrative costs compared with the potential benefits for continuity of care and stress reduction.

 In the medium term, psychosocial and community support* should be institutionalised* rather than left to sporadic charitable initiatives. Sustained community-based interventions, which are delivered through local organisations, schools, and volunteer networks, can provide psychological first aid, family-focused support, and activities that rebuild social cohesion.^18^ While these programmes require coordinated funding and cross-sector collaboration, they can be integrated into existing social service and community health infrastructures to enhance sustainability.

 In the long term, health systems should establish *structured post-disaster services and surveillance for affected residents and responders*, including respiratory, cardiovascular, and mental health assessment, rehabilitation, and long-term monitoring, with particular attention to displaced and high-risk individuals. Collaboration with academic institutions can help extend expertise, support evidence-based interventions, and generate data for service planning through sustained investment and governance oversight are required.

 Finally, *governance reforms* are essential to strengthening regulation and enforcement across the building lifecycle. Such reforms require coordination among government units, industry stakeholders, and the public,^19^ supported by transparent reporting mechanisms, regular fire safety education, and routine drills, particularly before major renovation works. Although these measures entail regulatory and administrative costs, stronger governance provides long-term returns by reducing preventable risks, improving accountability, and supporting sustainable urban safety.

## Disclosure of artificial intelligence (AI) use

 Not applicable.

## Ethical issues

 Not applicable.

## Conflicts of interest

 Authors declare that they have no conflicts of interest.
